# Molecular Basis for the Activation of Human Innate Immune Response by the Flagellin Derived from Plant-Pathogenic Bacterium, *Acidovorax avenae*

**DOI:** 10.3390/ijms22136920

**Published:** 2021-06-28

**Authors:** Nasir Javaid, Hiroyuki Hirai, Fang-Sik Che, Sangdun Choi

**Affiliations:** 1 Department of Molecular Science and Technology, Ajou University, Suwon 16499, Korea; 2 Graduate School of Bio-Science, Nagahama Institute of Bio-Science and Technology, 1266, Tamura Nagahama, Shiga 526-0829, Japan; h_hirai@nagahama-i-bio.ac.jp (H.H.); k_sai@nagahama-i-bio.ac.jp (F.-S.C.); 3 S&K Therapeutics, Woncheon Hall 135, Ajou University, Suwon 16499, Korea

**Keywords:** *Acidovorax avenae*, flagellin, TLR5, NLRC4

## Abstract

*Acidovorax avenae* is a flagellated, pathogenic bacterium to various plant crops that has also been found in human patients with haematological malignancy, fever, and sepsis; however, the exact mechanism for infection in humans is not known. We hypothesized that the human innate immune system could be responsive to the purified flagellin isolated from *A. avenae*, named FLA-AA. We observed the secretion of inflammatory cytokines such as tumor necrosis factor-alpha (TNF-α), interleukin (IL)-6, and IL-8 by treating FLA-AA to human dermal fibroblasts, as well as macrophages. This response was exclusively through TLR5, which was confirmed by using TLR5-overexpression cell line, 293/hTLR5, as well as TLR5-specific inhibitor, TH1020. We also observed the secretion of inflammatory cytokine, IL-1β, by the activation of NLRC4 with FLA-AA. Overall, our results provide a molecular basis for the inflammatory response caused by FLA-AA in cell-based assays.

## 1. Introduction

*Acidovorax avenae* is an oxidase-positive, non-lactose-fermenting, aerobic, rod-shaped, nonpigmented gram-negative bacterium that is pathogenic to various economically important crops such as rice, millet, maize, foxtail, sugarcane, and oats [1,2]. It is responsible for a devastating rice disease called bacterial brown stripe in some areas of Africa, Asia, Europe, and North America [3,4]. Although this species has done a lot of damage to plants, little is known about its opportunistic infection in humans. PCR amplification has confirmed the presence of *A. avenae* in a patient with a hematological cancer and fever [5]. In following year, *A. avenae* was identified in a sepsis patient by whole-cell long-chain-fatty-acid analysis [6]. An *A. avenae*-associated sepsis case related to an implanted-port-catheter has similarly been documented [7]. Another species, *A. oryzae*, has also been reported to cause bacteremia in humans in conjunction with a catheter [8]. Initially, *Acidovorax* spp. were classified as *Pseudomonas* spp.; however, their role as an animal or human pathogen has not been investigated in depth [9].

Pathogens possess certain signature molecules called “pathogen-associated molecular patterns” (PAMPs), which are recognized by host pattern recognition receptors (PRRs), such as Toll-like receptors (TLRs), to trigger the innate immune system in humans [10,11]. TLRs are type I transmembrane proteins with extracellular, transmembrane, and intracellular domains that recognize specific PAMPs with the help of leucine-rich repeats in their extracellular domain [12]. These TLRs are located either on the cell surface or in the membrane of endosomes or lysosomes to interact with their respective ligands [12]. TLRs are activated by diverse bacterial products: peptidoglycan, lipoarabinomannan, and bacterial lipoproteins interact with TLR2 [13,14]; lipopolysaccharide (LPS) mainly engages TLR4 [15,16]; and bacterial flagellin is recognized by TLR5 [17]. Endosomal TLRs recognize different sets of ligands: double-stranded RNA is recognized by TLR3 [18], single-stranded RNA is detected by TLR7 and TLR8 [19,20,21], and TLR9 interacts with viral and bacterial CpG DNA motifs and malarial pigment hemozoin [22,23,24]. The ligand recognition causes their Toll/interleukin-1 receptor (TIR) domains to interact with TIR domain-containing cytosolic adaptor molecules, thereby eventually leading to the activation of nuclear factor kappa light-chain-enhancer of activated B cells (NF-κB), of activating protein 1 (AP-1), and of interferon-regulatory factors (IRFs) [25,26]. These transcription factors regulate the expression of multiple cytokines that prepare the host against invaders. Nonetheless, improper activation of TLRs may result in various health problems such as cancer, rheumatoid arthritis, and sepsis.

Certain bacteria move with the help of a flagellum mainly composed of flagellin, which is a potent proinflammatory protein in both plants and humans that triggers the host defense system [17,27,28]. This protein possesses an evolutionarily conserved site that is responsible for bacterial motility and protofilament formation, and is recognized by TLR5 expressed on various cells, including intestinal epithelial cells and dendritic cells [17,29,30]. PAMP-primed cells activate NOD-like receptor (NLR) family CARD domain-containing protein 4 (NLRC4) upon detecting cytosolic flagellin during infection; this protein causes caspase 1 to release inflammatory cytokines like IL-1β and IL-18, and drives pyroptosis [31,32,33,34].

Species specificity of TLR5 to various bacterial flagellins has been demonstrated in its functional comparative analysis among humans, chickens, and mice [35,36]. These data prompted us to hypothesize that flagellin of plant-pathogenic bacteria provokes an immune response by interacting with human TLR5 and NLRC4.

## 2. Results

### 2.1. Activation of Immune Signaling in Human Macrophages

**Figure 1:**
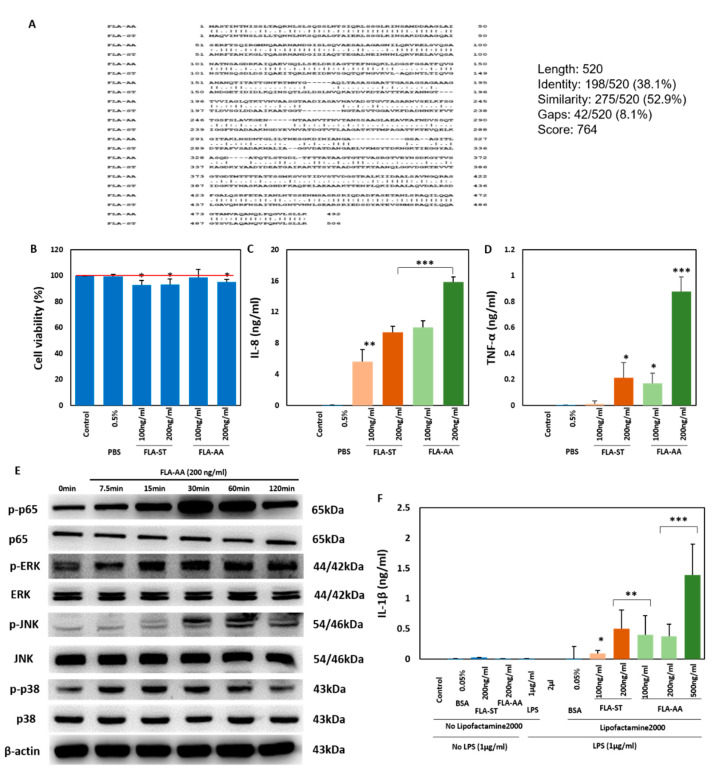
FLA-AA activates immune signaling in human macrophages. (**A**) The protein sequences of flagellin derived from FLA-AA and FLA-ST were compared via a pairwise local sequence alignment tool of EMBL-EBI, known as “EMBOSS water,” to assess the similarity between them. (**B**) Viability of THP-1 macrophages was determined by the MTT assay after the cells were treated with FLA-AA or FLA-ST at the indicated concentrations for 24 h. (**C**,**D**) THP-1 macrophages were treated with the indicated concentrations of FLA-AA or FLA-ST for 24 h; the culture supernatant was collected and processed for the quantitation of cytokines including IL-8 (**C**) and TNF-α (**D**) using respective ELISA kits. (**E**) Western blotting was performed to analyze the activation of NF-κB and MAPKs after treatment of THP-1 cells with FLA-AA (200 ng/mL) for the indicated periods. β-Actin served as an endogenous control. (**F**) PMA-differentiated THP-1 cells were primed with LPS for 4 h and transfected with FLA-AA or FLA-ST along with controls at the indicated concentrations for 2 h. The culture supernatant was analyzed for the secretion of IL-1β by means of a relevant ELISA kit. All the experiments were independently conducted four times, and differences between means ± SEM of the experimental groups were evaluated by two-tailed paired Student’s *t* test (* *p* < 0.05, ** *p* < 0.01, *** *p* < 0.001). FLA-AA: flagellin from *A. avenae*; FLA-ST: flagellin from *S. typhimurium*; IL-8: interleukin 8; LPS: lipopolysaccharide; MAPKs: mitogen-activated protein kinases; NF-κB: nuclear factor “kappa-light-chain-enhancer” of activated B cells; PMA: phorbol 12-myristate 13-acetate; TNF-α: tumor necrosis factor α.

Before functional analysis of FLA-AA, we checked its sequence similarity to the flagellin protein derived from *Salmonella typhimurium*, which has been reported to activate the human immune system via TLR5 [17] and NLRC4 [37]. We found 38.1% identity between their sequences by using the pairwise local sequence alignment tool of EMBL-EBI, “EMBOSS water” (Figure 1A). PAMPs are recognized by various host PRRs, such as TLRs, which allow them to dimerize for subsequent activation of downstream signaling pathways. The launched signaling pathways drive the secretion of various cytokines, such as tumor necrosis factor α (TNF-α), interleukin 6 (IL-6), and interleukin 8 (IL-8) [38]. Here, we evaluated the toxicity and immunogenicity of FLA-AA as well as FLA-ST (positive control) in relation to the activation of immune signaling pathways in PMA-differentiated human macrophages (THP-1 cells). The toxicity profile of FLA-AA was consistent with that of FLA-ST for THP-1 cells (toxic at higher concentrations; Figure 1B). We observed dose-dependent secretion of IL-8 (Figure 1C) and TNF-α (Figure 1D) by THP-1 cells after 24 h treatment. The recognition of a ligand by a TLR activates downstream NF-κB and mitogen-activated protein kinases (MAPKs), including extracellular signal-regulated kinase (ERK), c-Jun N-terminal kinase (JNK), and p38 [38]. In accordance with this result, we observed the activation of NF-κB (phosphorylation of p65) and MAPKs (phosphorylation of ERK, JNK, and p38) after treating the human macrophages (THP-1 cells) with FLA-AA in a time-dependent manner (Figure 1E). The cells primed with PAMPs activate NLRC4 upon recognition of the intracellular flagellin protein, which helps caspase 1 to release inflammatory cytokines such as IL-1β and IL-18 [31,32]. Because of the ability of the flagellin protein to activate NLRC4, we tested whether flagellin derived from the plant pathogen (FLA-AA) can stimulate NLRC4 in human cells, just like the one derived from human pathogens (FLA-ST, for instance). For this purpose, we utilized Lipofectamine 2000 for intracellular delivery of FLA-AA or FLA-ST, and noted significant secretion of IL-1β in a dose-dependent manner with the mixture of FLA-AA and Lipofectamine 2000; however, no IL-1β was detected upon treatment with FLA-AA alone (Figure 1F). Collectively, these data suggested that FLA-AA is immunogenic in terms of launching the TLR signaling pathway, as well as triggering NLRC4 upon treatment of the macrophages.

### 2.2. Secretion of Inflammatory Cytokines by Activated Primary Human Dermal Fibroblasts (HDFs)

**Figure 2:**
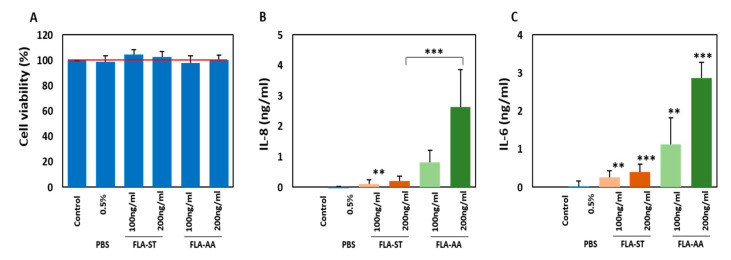
FLA-AA-activated HDFs secrete inflammatory cytokines: (**A**) Viability of HDFs was determined by the MTT assay after the cells were treated with FLA-AA or FLA-ST at the indicated concentrations for 24 h. (**B**,**C**) HDFs were treated with the indicated concentrations of FLA-AA or FLA-ST for 24 h; the culture supernatant was collected and processed for the quantitation of IL-8 (**B**) and IL-6 (**C**) using respective ELISA kits. All the experiments were independently conducted four times, and differences between means ± SEM of the experimental groups were evaluated by two-tailed paired Student’s *t* test (** *p* < 0.01, *** *p* < 0.001).

The skin immune system is composed of well-organized arrays of hematopoietic and non-hematopoietic cells that activate immune cells and sustain an equilibrium between pathogen defense and microbiome homeostasis. HDFs respond to pathogens by secreting multiple immune modulators such as IFN-β [39]. Hence, they can contribute to the establishment of pro-inflammatory microenvironment in the skin. Here, we evaluated the toxicity and immunogenicity of FLA-AA as well as FLA-ST on HDFs. The toxicity profile of FLA-AA was consistent with that of FLA-ST for HDFs (non-toxic; Figure 2A). We detected dose-dependent secretion of IL-8 (Figure 2B) and IL-6 (Figure 2C) by HDFs after treatment with FLA-AA or FLA-ST for 24 h.

### 2.3. FLA-AA–Mediated Signaling Is Specific to TLR5

**Figure 3:**
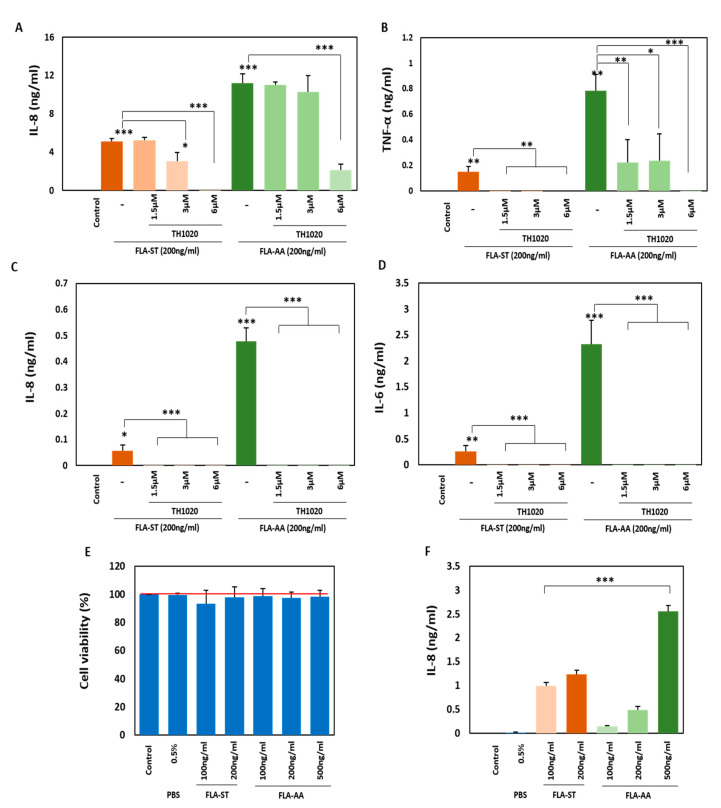
FLA-AA-mediated signaling is specific to TLR5: (**A**–**D**) THP-1 cells (10^5^/well) (**A**,**B**) and HDFs (10^4^/well) (**C**,**D**) were pre-treated for 1 h with various concentrations (1.5, 3, 6 µM) of TH1020 and post-treated with indicated concentration of FLA-ST and FLA-AA. After 24 h, the supernatant from THP-1 treated cells was used to calculate the level of IL-8 (A, C), TNF-α (**B**), and IL-6 (**D**) by ELISA experiment. (**E**) Cell viability on 293/hTLR5 (3 × 10^4^/well) was determined by MTT assay after treating them with FLA-AA and FLA-ST at mentioned concentrations for 24 h. (**F**) The secretion level of IL-8 in 293/hTLR5 cells was determined by ELISA experiment after 24 h of treatment with indicated concentrations of FLA-AA and FLA-ST. The statistical experiments were independently conducted four times and mean ± SEM of the experiments were evaluated in two-tailed paired Student’s *t* test (* *p* < 0.05, ** *p* < 0.01, *** *p* < 0.001).

The main building block of bacterial flagella, flagellin, has an evolutionarily conserved site that is recognized by TLR5 expressed on various cells [17,30]. A small molecule, TH1020, competes with flagellin for its binding to TLR5 and thus acts as a TLR5 inhibitor [40]. To evaluate the specificity of FLA-AA to TLR5 on THP-1 cells (Figure 3A,B) and HDFs (Figure 3C,D), we treated the cells with various concentrations of TH1020 (1.5, 3, or 6 µM) along with FLA-AA (200 ng/mL) or FLA-ST (200 ng/mL), and examined the concentrations of secreted cytokines. A TH1020-dependent reduction in the secretion of IL-8 (Figure 3A) and TNF-α (Figure 3B) was observed for THP-1 cells, while a reduction in the secretion of IL-8 (Figure 3C) and IL-6 (Figure 3D) was found for HDFs. Next, we evaluated FLA-AA toxicity and activation of signaling in a TLR5-overexpressing cell line (293/hTLR5). There was no significant toxicity during FLA-AA treatment up to 500 ng/mL (Figure 3E), and significant secretion of IL-8 (Figure 3F) was seen after 24 h of treatment.

**Figure 4:**
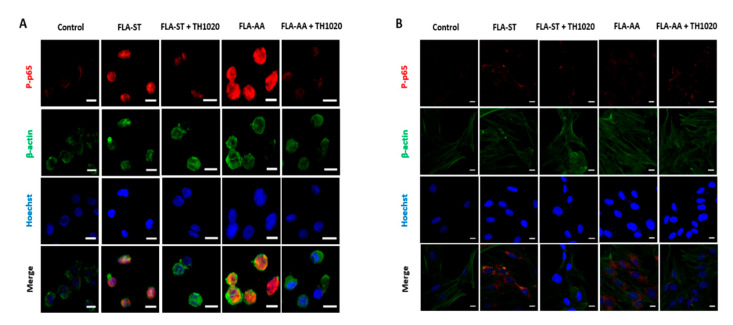
The immunofluorescent assay shows specificity of FLA-AA to TLR5: (**A**,**B**) THP-1 macrophages (**A**) and HDFs (**B**) were grown overnight in a 24-well plate. Then, the cells were pretreated for 1 h with the indicated concentration of TH1020. The post-treatment was based on FLA-AA or FLA-ST and was followed by the immunofluorescent assay with specific anti-p-p65 and anti-β-actin primary antibodies and Alexa Fluor-conjugated secondary antibodies. The red color indicates a phosphorylated subunit of NF-kB (p-p65); the green color denotes β-actin; and the blue color of the Hoechst 33,258 dye indicates nuclei. The images were captured at 40× magnification by means of an inverted microscope (Olympus IX53; Olympus Corporation, Tokyo, Japan). Scale bar: 50 μM.

The activation of TLR5 signaling leads to the phosphorylation of the p65 subunit of NF-κB, which can serve as an intracellular marker [38]. We visualized the phosphorylation of p65 (p-p65) via immunofluorescence after incubating FLA-AA (200 ng/mL) with THP-1 cells (Figure 4A) and HDFs (Figure 4B). As with previous results, we noted a reduction in fluorescence intensity of p-p65 after cotreating both cells with FLA-AA and the TLR5 inhibitor TH1020. These results confirmed that FLA-AA is specifically recognized by TLR5 to launch the downstream signaling pathway.

## 3. Discussion

The Gram-negative bacteria *S. enterica* and *Escherichia coli* are considered as model organisms to study flagellum, flagellar motility, and assembly. They possess a peritrichous pattern with five to six flagellar filaments over their cell body [41], and this number can vary based upon the cell cycle, swarming, and environmental conditions [42,43,44]. However, the increase in flagellar number during swarming motility and/or surface contact has not been reported for *Salmonella* [45]. The flagellum can affect virulence by bacterial adhesion, invasion, biofilm formation, survival, motility, secreting virulence factors, and triggering innate and adaptive immune response [17,46,47,48,49,50]. The flagellar motility acts as a virulence factor in multiple Gram-negative pathogens including *S. typhimurium*, *E. coli*, *Vibrio cholera*, and *Pseudomonas aeruginosa* [51,52,53,54,55]. However, bacteria lacking flagella can still be virulent, which indicates that flagella are not the only virulence factor to cause a disease [56,57]. A particular number of bacteria are required to cause an active infection in 50% of inoculated animals, which is represented by the term median infectious dose (ID_50_). The ID_50_ for *S. typhimurium*, *Shigella dysenteriae*, and *E. coli* are 1, 10–200, and 200–5000, respectively. However, the real infective dose for a particular individual can vary based upon the factors including immune status, age, and health of individual; route of entry; and environmental factors such as pH [58].

Facultative or opportunistic pathogenic bacteria cause a disease in a distant host after getting into that host when it is in a severely immunocompromised and debilitated condition [59,60]. Among them, a special group of bacteria causes hospital-acquired diseases known as nosocomial infections. For example, in one study, 45% of patients in intensive care units were reported to be infected with opportunistic pathogens in Europe [61]. *A. avenae* is a plant-pathogenic bacterium that infects various crops such as rice, maize, sugarcane, and oats [1,2]. This species causes a destructive rice disease in Asia, Africa, America, and Europe [3,4]. Aside from plants, it has been found in human patients with a fever, hematological cancer, sepsis, and other bloodstream infections [5,6,7,8]. So far, its immunomodulatory behavior toward humans has not been investigated at the molecular level.

We have previously reported the induction of PAMP-triggered immunity in plants by the flagellin isolated from a rice-avirulent strain, *A. avenae* N1141 (FLA-AA) [62]. Its sequence similarity with that of human pathogenic bacteria prompted us to hypothesize that purified flagellin can induce immune signaling at cellular and molecular levels in humans during an opportunistic infection. PAMPs are initially recognized by the PRRs of the innate immune system, and TLRs are some of the PRRs that can initiate TLR signaling by recognizing a cognate ligand [38]. Among extracellular TLRs, TLR5 is a homodimer that recognizes the flagellin protein of pathogenic-bacteria flagella [17,30]. The ligand recognition allows TLRs to interact with cytosolic adaptor molecules and downstream signaling molecules for the activation of such transcription factors as NF-κB, AP-1, and IRFs [25,26]. In the present study, we found out that FLA-AA has antigenic properties toward human macrophages and fibroblasts at the molecular level in terms of the secretion of proinflammatory cytokines. We next confirmed the specificity of FLA-AA by using a TLR5 inhibitor (TH1020) and a TLR5-overexpressing cell line (293/hTLR5), and found that the FLA-AA-induced signaling is mediated by TLR5. Moreover, immunofluorescent-assay results also supported the TLR5-mediated activation of signaling pathways by FLA-AA.

The inflammasome is a multiprotein cytoplasmic complex found in macrophages and dendritic cells, and activates caspase 1 to induce inflammatory-cell death (called pyroptosis) and a release of mature IL-1β and IL-18 [63]. Various cytoplasmic PRRs mediate assembly of inflammasomes and subsequent inflammasome-based innate immunity [33]. Among them, NLRC4 is a member of the NLRC subfamily of NLRs that is reported to recognize bacterial flagellin [31,32]. The bacterial flagellin protein contains two evolutionarily conserved leucine residues at its C terminus, as is common for many substrates of the type IV secretion system [64]. These collectively contribute to cytoplasmic delivery of flagellin for the activation of caspase 1. It has been reported that the NLRC4 inflammasome can be triggered either by infection or by purified flagellin from mammalian pathogenic bacteria such as *S. typhimurium* [65,66], *Legionella pneumophila* [32], *Pseudomonas aeruginosa* [67], and *Listeria monocytogenes* [68]. In our study, purified flagellin from a plant-pathogenic bacterium, *A. avenae*, activated caspase 1 for the release of mature IL-1β.

The FLA-AA showed in vitro efficacy within the concentration range (1 to 200 ng/mL) tested for flagellin purified from other bacterial strains [69,70,71,72,73]. This correlation suggests a similar in vivo efficacy at the doses (1 to 30 μg) tested for flagellin from other sources [50,74,75,76,77]. However, the improper activation of a particular TLR or a group of TLRs is associated with various diseases. For example, TLR2 is associated with systemic lupus erythematosus (SLE) [78], sepsis [79], psoriasis [80]; TLR3 with sepsis [81]; TLR4 with SLE [82], sepsis [83], and psoriasis [80]; TLR5 with psoriasis [80]; TLR7 with SLE [84], stroke [85], and psoriasis [86]; and TLR9 is involved in SLE [87], sepsis [88] and psoriasis [89]. Hence, it is important to understand the molecular mechanism of disease progression by a particular pathogen in association to innate immune receptors. Our finding confirmed it for the flagellin purified from *A. avenae*, which might help to cure associated inflammatory diseases by inhibiting the pathway.

## 4. Materials and Methods

### 4.1. Sequence Alignment

The protein databank of National Center for Biotechnology Information (NCBI, Bethesda, MD, USA) was used to retrieve the full-length sequence of FLA-ST (https://www.ncbi.nlm.nih.gov/protein/KYJ52374.1, accessed data on 2 August 2020) and FLA-AA (https://www.ncbi.nlm.nih.gov/protein/WP_107178774.1, accessed data on 2 August 2020). The similarity between them was analyzed with Smith–Waterman algorithm by using the pairwise sequence alignment tool, EMBL-EBI (EMBOSS water) (https://www.ebi.ac.uk/Tools/psa/emboss_water/, Hinxton, Cambridgeshire, UK, accessed data on 2 August 2020).

### 4.2. Cell Lines and Reagents

THP-1 cells (kindly gifted by Dr. Chang-Hee Suh, Ajou University, Medical Center, Suwon, Korea) were cultured in the RPMI 1640 medium (Gibco, BRL, Grand Island, NY, USA), supplemented with 1% of a penicillin/streptomycin solution (HyClone; Cytiva, Marlborough, MA, USA), and 10% of heat-inactivated fetal bovine serum (FBS; Thermo Fisher Scientific, Inc., Waltham, MA, USA). THP-1 cells were differentiated into macrophages using 80 nM phorbol 12-myristate 13-acetate (PMA; Sigma–Aldrich Co., St. Louis, MO, USA) for 24 h. Human dermal fibroblasts (HDFs; ATCC, Manassas, VA, USA) and 293/hTLR5 cell line ((InvivoGen, San Diego, CA, USA) were cultured in high-glucose Dulbecco’s modified Eagle’s medium (DMEM, Gibco, BRL, Grand Island, NY, USA) containing 1% of the penicillin/streptomycin solution (HyClone; Cytiva, Marlborough, MA, USA), 10% of FBS (Thermo Fisher Scientific, Inc., Waltham, MA, USA), and 0.2% of Normocin (InvivoGen, San Diego, CA, USA). All the cells were incubated in a humidified atmosphere containing 5% of CO_2_ at 37 °C (Thermo Fisher Scientific, Inc., Waltham, MA, USA). Lipofectamine 2000 was purchased from Thermo Fisher Scientific, Inc. (Waltham, MA, USA); FLA-ST from InvivoGen (San Diego, CA, USA); and TH1020 from Tocris (Cookson, Bristol, UK). FLA-AA (flagellin from *A. avenae*) was purified according to a previously described protocol [62].

### 4.3. Cell Viability Assay

A colorimetric 1-(4,5-dimethylthiazol-2-yl)-3,5-diphenylformazan (MTT) assay (Sigma–Aldrich Co., St. Louis, MO, USA) was performed to calculate cell viability. THP-1 cells (10^5^/well) and HDFs (10^4^/well) were seeded and grown overnight in 96-well plates (BD Biosciences, San Jose, CA, USA). The cells were treated with various concentrations of FLA-ST or FLA-AA for 24 h. The next day, the medium was replaced with a 10% MTT solution in the medium (100 µL/well) and incubated for 3 h at 37 °C. It was replaced with DMSO (Sigma-Aldrich Corp., St. Louis, MO, USA; 100 µL/well), and the plates were incubated at 37 °C for 30 min. The plates were read at 540 nm wavelength using a Synergy HTX multi-mode reader (Bio-Tek, Winooski, VT, USA).

### 4.4. Western Blot Analyses

The M-PER Mammalian Protein Extraction Reagent (Thermo Fisher Scientific, Inc., Waltham, MA, USA) was employed to extract total cellular protein, and protein concentration was measured with the Bicinchoninic Acid (BCA) Assay Kit (Sigma–Aldrich Waltham, MA, USA). The protein samples were electrophoresed and transferred to a membrane in a Mini-PROTEAN Tetra Cell and Mini Trans-Blot Electrophoretic Transfer Cell System (Bio-Rad Laboratories, Hercules, CA, USA). The membranes were immunoblotted with specific primary antibodies (dilution 1:1000) against phospho- (p-)p65, p65, ERK, JNK, p-JNK, p38, p-p38, Iκ-Bα (Cell Signaling Technology Inc., Danvers, MA, USA), p-ERK, and β-actin (Santa Cruz Biotechnology Inc., Dallas, TX, USA), with gentle shaking at 4 °C overnight. After rigorous washing with phosphate buffered saline (PBS; VWR International, Radnor, PA, USA) supplemented with 0.1% of Tween 20 (Anatrace Inc., Maumee, OH, USA), the membranes were incubated with a peroxidase-conjugated anti-mouse or anti-rabbit IgG antibody (Thermo Fisher Scientific, Inc., Waltham, MA, USA; 1:1000) at room temperature for 2 h. The protein bands were detected by means of a SuperSignal West Pico ECL Solution (Thermo Fisher Scientific, Inc., Waltham, MA, USA) and visualized using a ChemiDoc™ Touch Imaging System (Bio-Rad Laboratories; Hercules, CA, USA).

### 4.5. Immunofluorescence

THP-1 cells and HDFs were grown on coverslips at a density of 10^4^/well in 24-well plates (BD Biosciences). The cells were either pretreated or not pretreated with TH1020 (3 μM) for 1 h before the treatment with FLA-ST or FLA-AA (200 ng/mL). After that, the cells were fixed and permeabilized in chilled methanol (Samchun Chemicals, Korea) for 10 min, washed with PBS, and blocked with a 3% BSA (Biosesang, Seongnam, Gyonggi, Korea) solution in PBS for 30 min. The cells were incubated with the anti-p-p65 antibody (1:1000; Cell Signaling Technology Inc., Danvers, MA, USA) and the anti-β-actin antibody (1:1000; Santa Cruz Biotechnology Inc., Dallas, TX, USA) at 4 °C overnight. After rigorous washing, the cells were incubated with an Alexa Fluor 488- or Alexa Fluor 546-conjugated secondary antibody (Invitrogen, Carlsbad, CA, USA) for 1 h at room temperature. After a wash with PBS, nuclei were stained with a Hoechst 33,258 solution (5 µM; Sigma-Aldrich Corp., St. Louis, MO, USA) for 10 min. Images were captured using a fluorescence microscope (Olympus IX53; Olympus Corporation, Tokyo, Japan).

### 4.6. Cytokine Detection Assays

THP-1 derived macrophage cells (10^5^/well), HDFs (10^4^/well), and 293/hTLR5 cells (3 × 10^4^/well) were seeded and grown overnight in 96-well plates (BD Biosciences). After 24 h of treatment, TNF-α production was assessed by means of the Human TNF Alpha Uncoated ELISA Kit (eBioscience, San Diego, CA, USA); IL-6 by the Human IL-6 ELISA MAX Deluxe Kit (BioLegend, San Diego, CA, USA); IL-8 by the Human IL-8 Uncoated ELISA Kit (Thermo Fisher Scientific, Inc., Waltham, MA, USA). For NLRC4 activation, THP-1 macrophages were primed with LPS (InvivoGen, San Diego, CA, USA; 1 μg/mL) for 4 h, followed by transfection of FLA-AA and FLA-ST via Lipofectamine 2000 for 2 h. The culture supernatant was collected to assess the secreted amount of IL-1β with the IL-1 beta Human Uncoated ELISA Kit (Thermo Fisher Scientific, Inc., Waltham, MA, USA). The plates were then analyzed on a Synergy HTX multi-mode reader (Bio-Tek, Winooski, VT, USA) at respective wavelengths.

### 4.7. Statistical Analysis

The presented data are averages of four independent experiments, and differences between means ± SEM of the experimental groups were evaluated by two-tailed paired Student’s *t* test in Microsoft Excel 2016 (Microsoft Corp., Redmond, WA, USA; * *p* < 0.05, ** *p* < 0.01, *** *p* < 0.001).

## 5. Conclusions

Some pathogens can infect the host of a different kingdom just as a plant-pathogenic bacterium, such as *A. avenae,* can also cause infection in humans. One of the virulence factors of *A. avenae* is purified flagellin protein, which can activate the innate immune receptors of human macrophages as well as fibroblasts. Their activation leads to the secretion of cytokines, which can result in various inflammatory diseases. This molecular understanding could be beneficial in cures by inhibiting the involved signaling pathways.

## Data Availability

The data presented in this study are available in the main article.

## References

[1] Song W., Kim H., Hwang C., Schaad N. (2004). Detection of *Acidovorax avenae* ssp. avenae in Rice Seeds Using BIO-PCR. J. Phytopathol..

[2] Lipuma J.J., Currie B.J., Peacock S.J., Vandamme P.A. (2015). Burkholderia, Stenotrophomonas, Ralstonia, Cupriavidus, Pandoraea, Brevundimonas, Comamonas, Delftia, andAcidovorax. Manual of Clinical Microbiology.

[3] Xie G., Sun X., Mew W. (1998). Characterization of *Acidovorax avenae* subsp. avenae from rice seeds. Zhongguo Shuidao Kexue.

[4] Xie G.-L., Zhang G.-Q., Liu H., Lou M.-M., Tian W.-X., Li B., Zhou X.-P., Zhu B., Jin G.-L. (2011). Genome sequence of the rice-pathogenic bacterium *Acidovorax avenae* subsp. avenae RS-1. J. Bacteriol..

[5] Xu J., Moore J.E., Millar B.C., Alexander H.D., McClurg R., Morris T.C., Rooney P.J. (2004). Improved laboratory diagnosis of bacterial and fungal infections in patients with hematological malignancies using PCR and ribosomal RNA sequence analysis. Leuk. Lymphoma.

[6] Shetty A., Barnes R., Healy B., Groves P. (2005). A case of sepsis caused by Acidovorax. J. Infect..

[7] Malkan A.D., Strollo W., Scholand S.J., Dudrick S.J. (2009). Implanted-port-catheter-related sepsis caused by *Acidovorax avenae* and methicillin-sensitive Staphylococcus aureus. J. Clin. Microbiol..

[8] Orsborne C., Hardy A., Isalska B., Williams S.G., Muldoon E.G. (2014). Acidovorax oryzae catheter-associated bloodstream infection. J. Clin. Microbiol..

[9] Willems A., Falsen E., Pot B., Jantzen E., Hoste B., Vandamme P., Gillis M., Kersters K., De Ley J. (1990). Acidovorax, a new genus for *Pseudomonas facilis*, *Pseudomonas delafieldii*, E. Falsen (EF) group 13, EF group 16, and several clinical isolates, with the species *Acidovorax facilis* comb. nov., *Acidovorax delafieldii* comb. nov., and *Acidovorax temperans* sp. nov. Int. J. Syst. Evol. Microbiol..

[10] Janeway C.A. (1989). Approaching the asymptote? Evolution and revolution in immunology. Cold Spring Harb. Symp. Quant. Biol..

[11] Medzhitov R., Preston-Hurlburt P., Janeway C.A. (1997). A human homologue of the Drosophila Toll protein signals activation of adaptive immunity. Nature.

[12] Uematsu S., Akira S. (2008). Toll-Like receptors (TLRs) and their ligands. Toll-Like Receptors (TLRs) and Innate Immunity.

[13] Lien E., Sellati T.J., Yoshimura A., Flo T.H., Rawadi G., Finberg R.W., Carroll J.D., Espevik T., Ingalls R.R., Radolf J.D. (1999). Toll-like receptor 2 functions as a pattern recognition receptor for diverse bacterial products. J. Biol. Chem..

[14] Brightbill H.D., Libraty D.H., Krutzik S.R., Yang R.-B., Belisle J.T., Bleharski J.R., Maitland M., Norgard M.V., Plevy S.E., Smale S.T. (1999). Host defense mechanisms triggered by microbial lipoproteins through toll-like receptors. Science.

[15] Poltorak A., He X., Smirnova I., Liu M.-Y., Van Huffel C., Du X., Birdwell D., Alejos E., Silva M., Galanos C. (1998). Defective LPS signaling in C3H/HeJ and C57BL/10ScCr mice: Mutations in Tlr4 gene. Science.

[16] Chow J.C., Young D.W., Golenbock D.T., Christ W.J., Gusovsky F. (1999). Toll-like receptor-4 mediates lipopolysaccharide-induced signal transduction. J. Biol. Chem..

[17] Hayashi F., Smith K.D., Ozinsky A., Hawn T.R., Eugene C.Y., Goodlett D.R., Eng J.K., Akira S., Underhill D.M., Aderem A. (2001). The innate immune response to bacterial flagellin is mediated by Toll-like receptor 5. Nature.

[18] Alexopoulou L., Holt A.C., Medzhitov R., Flavell R.A. (2001). Recognition of double-stranded RNA and activation of NF-κB by Toll-like receptor 3. Nature.

[19] Diebold S.S., Kaisho T., Hemmi H., Akira S., e Sousa C.R. (2004). Innate antiviral responses by means of TLR7-mediated recognition of single-stranded RNA. Science.

[20] Heil F., Hemmi H., Hochrein H., Ampenberger F., Kirschning C., Akira S., Lipford G., Wagner H., Bauer S. (2004). Species-specific recognition of single-stranded RNA via toll-like receptor 7 and 8. Science.

[21] Jurk M., Heil F., Vollmer J., Schetter C., Krieg A.M., Wagner H., Lipford G., Bauer S. (2002). Human TLR7 or TLR8 independently confer responsiveness to the antiviral compound R-848. Nat. Immunol..

[22] Hemmi H., Takeuchi O., Kawai T., Kaisho T., Sato S., Sanjo H., Matsumoto M., Hoshino K., Wagner H., Takeda K. (2000). A Toll-like receptor recognizes bacterial DNA. Nature.

[23] Lund J., Sato A., Akira S., Medzhitov R., Iwasaki A. (2003). Toll-like receptor 9–mediated recognition of Herpes simplex virus-2 by plasmacytoid dendritic cells. J. Exp. Med..

[24] Coban C., Ishii K.J., Kawai T., Hemmi H., Sato S., Uematsu S., Yamamoto M., Takeuchi O., Itagaki S., Kumar N. (2005). Toll-like receptor 9 mediates innate immune activation by the malaria pigment hemozoin. J. Exp. Med..

[25] Ve T., Gay N.J., Mansell A., Kobe B., Kellie S. (2012). Adaptors in toll-like receptor signaling and their potential as therapeutic targets. Curr. Drug Targets.

[26] Gay N.J., Symmons M.F., Gangloff M., Bryant C.E. (2014). Assembly and localization of Toll-like receptor signalling complexes. Nat. Rev. Immunol..

[27] Gómez-Gómez L., Boller T. (2000). FLS2: An LRR receptor–like kinase involved in the perception of the bacterial elicitor flagellin in Arabidopsis. Mol. Cell.

[28] Bauer Z., Gómez-Gómez L., Boller T., Felix G. (2001). Sensitivity of different ecotypes and mutants ofArabidopsis thaliana toward the bacterial elicitor flagellin correlates with the presence of receptor-binding sites. J. Biol. Chem..

[29] Uematsu S., Jang M.H., Chevrier N., Guo Z., Kumagai Y., Yamamoto M., Kato H., Sougawa N., Matsui H., Kuwata H. (2006). Detection of pathogenic intestinal bacteria by Toll-like receptor 5 on intestinal CD11c+ lamina propria cells. Nat. Immunol..

[30] Gewirtz A.T., Navas T.A., Lyons S., Godowski P.J., Madara J.L. (2001). Cutting edge: Bacterial flagellin activates basolaterally expressed TLR5 to induce epithelial proinflammatory gene expression. J. Immunol..

[31] Miao E.A., Alpuche-Aranda C.M., Dors M., Clark A.E., Bader M.W., Miller S.I., Aderem A. (2006). Cytoplasmic flagellin activates caspase-1 and secretion of interleukin 1β via Ipaf. Nat. Immunol..

[32] Amer A., Franchi L., Kanneganti T.-D., Body-Malapel M., Özören N., Brady G., Meshinchi S., Jagirdar R., Gewirtz A., Akira S. (2006). Regulation of Legionella phagosome maturation and infection through flagellin and host Ipaf. J. Biol. Chem..

[33] Miao E.A., Leaf I.A., Treuting P.M., Mao D.P., Dors M., Sarkar A., Warren S.E., Wewers M.D., Aderem A. (2010). Caspase-1-induced pyroptosis is an innate immune effector mechanism against intracellular bacteria. Nat. Immunol..

[34] Cai S., Batra S., Wakamatsu N., Pacher P., Jeyaseelan S. (2012). NLRC4 inflammasome-mediated production of IL-1β modulates mucosal immunity in the lung against gram-negative bacterial infection. J. Immunol..

[35] Andersen-Nissen E., Smith K.D., Bonneau R., Strong R.K., Aderem A. (2007). A conserved surface on Toll-like receptor 5 recognizes bacterial flagellin. J. Exp. Med..

[36] Keestra A.M., de Zoete M.R., van Aubel R.A., van Putten J.P. (2008). Functional characterization of chicken TLR5 reveals species-specific recognition of flagellin. Mol. Immunol..

[37] Zhao Y., Yang J., Shi J., Gong Y.-N., Lu Q., Xu H., Liu L., Shao F. (2011). The NLRC4 inflammasome receptors for bacterial flagellin and type III secretion apparatus. Nature.

[38] Akira S., Takeda K. (2004). Toll-like receptor signalling. Nat. Rev. Immunol..

[39] Goodbourn S., Didcock L., Randall R. (2000). Interferons: Cell signalling, immune modulation, antiviral response and virus countermeasures. J. Gen. Virol..

[40] Yan L., Liang J., Yao C., Wu P., Zeng X., Cheng K., Yin H. (2016). Pyrimidine Triazole Thioether Derivatives as Toll-Like Receptor 5 (TLR5)/Flagellin Complex Inhibitors. ChemMedChem.

[41] Leifson E. (1960). Atlas of bacterial flagellation. Atlas of Bacterial Flagellation.

[42] Ping L. (2010). The asymmetric flagellar distribution and motility of Escherichia coli. J. Mol. Biol..

[43] Chilcott G.S., Hughes K.T. (2000). Coupling of flagellar gene expression to flagellar assembly in Salmonella enterica serovar typhimurium and Escherichia coli. Microbiol. Mol. Biol. Rev..

[44] Aizawa S.I., Kubori T. (1998). Bacterial flagellation and cell division. Genes Cells.

[45] Partridge J.D., Harshey R.M. (2013). More than motility: Salmonella flagella contribute to overriding friction and facilitating colony hydration during swarming. J. Bacteriol..

[46] Duan Q., Zhou M., Zhu X., Bao W., Wu S., Ruan X., Zhang W., Yang Y., Zhu J., Zhu G. (2012). The flagella of F18ab Escherichia coli is a virulence factor that contributes to infection in a IPEC-J2 cell model in vitro. Vet. Microbiol..

[47] Duan Q., Zhou M., Zhu X., Yang Y., Zhu J., Bao W., Wu S., Ruan X., Zhang W., Zhu G. (2013). Flagella from F18+ Escherichia coli play a role in adhesion to pig epithelial cell lines. Microb. Pathog..

[48] Arora S.K., Ritchings B.W., Almira E.C., Lory S., Ramphal R. (1996). Cloning and characterization of Pseudomonas aeruginosa fliF, necessary for flagellar assembly and bacterial adherence to mucin. Infect. Immun..

[49] Olsen J.E., Hoegh-Andersen K.H., Casadesús J., Rosenkrantz J.T., Chadfield M.S., Thomsen L.E. (2013). The role of flagella and chemotaxis genes in host pathogen interaction of the host adapted Salmonella enterica serovar Dublin compared to the broad host range serovar S. Typhimurium. BMC Microbiol..

[50] Crawford R.W., Reeve K.E., Gunn J.S. (2010). Flagellated but not hyperfimbriated Salmonella enterica serovar Typhimurium attaches to and forms biofilms on cholesterol-coated surfaces. J. Bacteriol..

[51] Allen-Vercoe E., Woodward M.J. (1999). The role of flagella, but not fimbriae, in the adherence of Salmonella enterica serotype Enteritidis to chick gut explant. J. Med Microbiol..

[52] Marchetti M., Sirard J.C., Sansonetti P., Pringault E., Kernéis S. (2004). Interaction of pathogenic bacteria with rabbit appendix M cells: Bacterial motility is a key feature in vivo. Microbes Infect..

[53] La Ragione R., Sayers A., Woodward M.J. (2000). The role of fimbriae and flagella in the colonization, invasion and persistence of Escherichia coli O78 [ratio] K80 in the day-old-chick model. Epidemiol. Infect..

[54] Krukonis E.S., DiRita V.J. (2003). From motility to virulence: Sensing and responding to environmental signals in Vibrio cholerae. Curr. Opin. Microbiol..

[55] Arora S.K., Neely A.N., Blair B., Lory S., Ramphal R. (2005). Role of motility and flagellin glycosylation in the pathogenesis of Pseudomonas aeruginosa burn wound infections. Infect. Immun..

[56] Lockman H.A., Curtiss R. (1990). Salmonella typhimurium mutants lacking flagella or motility remain virulent in BALB/c mice. Infect. Immun..

[57] Gardel C.L., Mekalanos J.J. (1996). Alterations in Vibrio cholerae motility phenotypes correlate with changes in virulence factor expression. Infect. Immun..

[58] Lampel K., Al-Khaldi S., Cahill S., Food and Drug Administration (2012). Bad bug book, foodborne pathogenic microorganisms and natural toxins. Gram-positiVe Bacteria.

[59] Parke J.L., Gurian-Sherman D. (2001). Diversity of the Burkholderia cepacia complex and implications for risk assessment of biological control strains. Annu. Rev. Phytopathol..

[60] Steinkamp G., Wiedemann B., Rietschel E., Krahl A., Gielen J., Bärmeier H., Ratjen F., Group E.B.S. (2005). Prospective evaluation of emerging bacteria in cystic fibrosis. J. Cyst. Fibros..

[61] Vincent J.-L., Bihari D.J., Suter P.M., Bruining H.A., White J., Nicolas-Chanoin M.-H., Wolff M., Spencer R.C., Hemmer M. (1995). The prevalence of nosocomial infection in intensive care units in Europe: Results of the European Prevalence of Infection in Intensive Care (EPIC) Study. JAMA.

[62] Hirai H., Furukawa T., Katsuragi Y., Che F.-S. (2016). Purification of Flagellin from *Acidovorax avenae* and Analysis of Plant Immune Responses Induced by the Purified Flagellin. Bio-Protocol.

[63] Martinon F., Burns K., Tschopp J. (2002). The inflammasome: A molecular platform triggering activation of inflammatory caspases and processing of proIL-β. Mol. Cell.

[64] Nagai H., Cambronne E.D., Kagan J.C., Amor J.C., Kahn R.A., Roy C.R. (2005). A C-terminal translocation signal required for Dot/Icm-dependent delivery of the Legionella RalF protein to host cells. Proc. Natl. Acad. Sci. USA.

[65] Franchi L., Amer A., Body-Malapel M., Kanneganti T.-D., Özören N., Jagirdar R., Inohara N., Vandenabeele P., Bertin J., Coyle A. (2006). Cytosolic flagellin requires Ipaf for activation of caspase-1 and interleukin 1β in salmonella-infected macrophages. Nat. Immunol..

[66] Sun Y.-H., Rolán H.G., Tsolis R.M. (2007). Injection of flagellin into the host cell cytosol by Salmonella enterica serotype Typhimurium. J. Biol. Chem..

[67] Miao E.A., Ernst R.K., Dors M., Mao D.P., Aderem A. (2008). Pseudomonas aeruginosa activates caspase 1 through Ipaf. Proc. Natl. Acad. Sci. USA.

[68] Warren S.E., Mao D.P., Rodriguez A.E., Miao E.A., Aderem A. (2008). Multiple Nod-like receptors activate caspase 1 during Listeria monocytogenes infection. J. Immunol..

[69] Ellenbroek G.H., Van Puijvelde G.H., Anas A.A., Bot M., Asbach M., Schoneveld A., Van Santbrink P.J., Foks A.C., Timmers L., Doevendans P.A. (2017). Leukocyte TLR5 deficiency inhibits atherosclerosis by reduced macrophage recruitment and defective T-cell responsiveness. Sci. Rep..

[70] Koosakulnirand S., Phokrai P., Jenjaroen K., Roberts R.A., Utaisincharoen P., Dunachie S.J., Brett P.J., Burtnick M.N., Chantratita N. (2018). Immune response to recombinant Burkholderia pseudomallei FliC. PLoS ONE.

[71] Song E.-J., Kang M.-J., Kim Y.-S., Kim S.-M., Lee S.-E., Kim C.-H., Kim D.-J., Park J.-H. (2011). Flagellin promotes the proliferation of gastric cancer cells via the Toll-like receptor 5. Int. J. Mol. Med..

[72] Orhan F., Bhat M., Sandberg K., Ståhl S., Piehl F., Consortium K.S.P., Svensson C., Erhardt S., Schwieler L., Farde L. (2016). Tryptophan Metabolism Along the Kynurenine Pathway Downstream of Toll—Like Receptor Stimulation in Peripheral Monocytes. Scand. J. Immunol..

[73] Weaver L.K., Hintz-Goldstein K.A., Pioli P.A., Wardwell K., Qureshi N., Vogel S.N., Guyre P.M. (2006). Pivotal advance: Activation of cell surface Toll-like receptors causes shedding of the hemoglobin scavenger receptor CD163. J. Leukoc. Biol..

[74] Zhou S.-X., Li F.-S., Qiao Y.-L., Zhang X.-Q., Wang Z.-D. (2012). Toll-like receptor 5 agonist inhibition of growth of A549 lung cancer cells in vivo in a Myd88 dependent manner. Asian Pac. J. Cancer Prev..

[75] Sfondrini L., Rossini A., Besusso D., Merlo A., Tagliabue E., Mènard S., Balsari A. (2006). Antitumor activity of the TLR-5 ligand flagellin in mouse models of cancer. J. Immunol..

[76] Rhee S.H., Im E., Pothoulakis C. (2008). Toll-like receptor 5 engagement modulates tumor development and growth in a mouse xenograft model of human colon cancer. Gastroenterology.

[77] Burdelya L.G., Gleiberman A.S., Toshkov I., Aygun-Sunar S., Bapardekar M., Manderscheid-Kern P., Bellnier D., Krivokrysenko V.I., Feinstein E., Gudkov A.V. (2012). Toll-like receptor 5 agonist protects mice from dermatitis and oral mucositis caused by local radiation: Implications for head-and-neck cancer radiotherapy. Int. J. Radiat. Oncol..

[78] Komatsuda A., Wakui H., Iwamoto K., Ozawa M., Togashi M., Masai R., Maki N., Hatakeyama T., Sawada K. (2008). Up-regulated expression of Toll-like receptors mRNAs in peripheral blood mononuclear cells from patients with systemic lupus erythematosus. Clin. Exp. Immunol..

[79] Bergt S., Wagner N.-M., Heidrich M., Butschkau A., Nöldge-Schomburg G.E., Vollmar B., Roesner J.P. (2013). Hydrocortisone reduces the beneficial effects of toll-like receptor 2 deficiency on survival in a mouse model of polymicrobial sepsis. Shock.

[80] Seung N.R., Park E.J., Kim C.W., Kim K.H., Kim K.J., Cho H.J., Park H.R. (2007). Comparison of expression of heat-shock protein 60, Toll-like receptors 2 and 4, and T-cell receptor γδ in plaque and guttate psoriasis. J. Cutan. Pathol..

[81] Cavassani K.A., Ishii M., Wen H., Schaller M.A., Lincoln P.M., Lukacs N.W., Hogaboam C.M., Kunkel S.L. (2008). TLR3 is an endogenous sensor of tissue necrosis during acute inflammatory events. J. Exp. Med..

[82] Liu B., Yang Y., Dai J., Medzhitov R., Freudenberg M.A., Zhang P.L., Li Z. (2006). TLR4 up-regulation at protein or gene level is pathogenic for lupus-like autoimmune disease. J. Immunol..

[83] Roger T., Froidevaux C., Le Roy D., Reymond M.K., Chanson A.-L., Mauri D., Burns K., Riederer B.M., Akira S., Calandra T. (2009). Protection from lethal gram-negative bacterial sepsis by targeting Toll-like receptor 4. Proc. Natl. Acad. Sci. USA.

[84] Pisitkun P., Deane J.A., Difilippantonio M.J., Tarasenko T., Satterthwaite A.B., Bolland S. (2006). Autoreactive B cell responses to RNA-related antigens due to TLR7 gene duplication. Science.

[85] Leung P.Y., Stevens S.L., Packard A.E., Lessov N.S., Yang T., Conrad V.K., Van Den Dungen N.N., Simon R.P., Stenzel-Poore M.P. (2012). Toll-like receptor 7 preconditioning induces robust neuroprotection against stroke by a novel type I interferon-mediated mechanism. Stroke.

[86] Gilliet M., Conrad C., Geiges M., Cozzio A., Thürlimann W., Burg G., Nestle F.O., Dummer R. (2004). Psoriasis triggered by toll-like receptor 7 agonist imiquimod in the presence of dermal plasmacytoid dendritic cell precursors. Arch. Dermatol..

[87] Jackson S.W., Scharping N.E., Kolhatkar N.S., Khim S., Schwartz M.A., Li Q.-Z., Hudkins K.L., Alpers C.E., Liggitt D., Rawlings D.J. (2014). Opposing impact of B cell–intrinsic TLR7 and TLR9 signals on autoantibody repertoire and systemic inflammation. J. Immunol..

[88] Hu D., Yang X., Xiang Y., Li H., Yan H., Zhou J., Caudle Y., Zhang X., Yin D. (2015). Inhibition of Toll-like receptor 9 attenuates sepsis-induced mortality through suppressing excessive inflammatory response. Cell. Immunol..

[89] Miller L.S., Sørensen O.E., Liu P.T., Jalian H.R., Eshtiaghpour D., Behmanesh B.E., Chung W., Starner T.D., Kim J., Sieling P.A. (2005). TGF-α regulates TLR expression and function on epidermal keratinocytes. J. Immunol..

